# The Promise and Challenges of Mesenchymal Stem Cell-Derived Extracellular Vesicles in Periodontal Disease

**DOI:** 10.3390/pathogens15040420

**Published:** 2026-04-13

**Authors:** Jonghoe Byun

**Affiliations:** Department of Biological Sciences/Department of Molecular Biology, Institute of Nanosensor and Biotechnology, Dankook University, Dandaero 119, Dongnam-gu, Cheonan-si 31116, Chungnam, Republic of Korea; jonghoe@dankook.ac.kr; Tel.: +82-41-550-3488

**Keywords:** periodontal disease, mesenchymal stem cell (MSC), paracrine factor, extracellular vesicles (EVs), immunomodulator

## Abstract

Periodontal disease represents a major global health burden, beginning with gingivitis and progressing to periodontitis, which causes connective tissue breakdown, alveolar bone resorption, and eventual tooth loss. Beyond local pathology, periodontitis is a chronic inflammatory condition with systemic associations, including cardiovascular disease, diabetes, and metabolic disorders. Mesenchymal stem cells (MSCs) and their extracellular vesicles (EVs) have emerged as promising candidates for periodontal regeneration. This review aimed to map the current evidence on MSC-derived EVs (MSC-EVs) in periodontal regeneration, focusing on their mechanisms of action, therapeutic potential, and translational challenges. A comprehensive literature search was conducted across a major biomedical database (PubMed) to identify preclinical and clinical studies investigating MSC-EVs in the context of periodontitis. Data were charted on EV cargo composition, biological functions, regenerative outcomes, and reported limitations. Evidence indicates that MSC-EVs encapsulate bioactive molecules—including antimicrobial peptides, proteins, lipids, and microRNAs—that modulate immune responses, suppress pro-inflammatory signaling, and promote angiogenesis and tissue repair. In periodontal models, MSC-EVs attenuate osteoclast activity, enhance fibroblast proliferation, and stimulate extracellular matrix remodeling, supporting regeneration of periodontal ligament and alveolar bone. Exosome-based approaches demonstrate advantages such as reduced immunogenicity, improved safety, and feasibility for storage and standardization. However, most findings remain preclinical, with limited human data available. To bridge the translational gap, well-designed clinical trials are needed to confirm efficacy and safety while addressing regulatory challenges, GMP standards, and outcome measures. Harnessing their regenerative capacity while mitigating side effects may guide precision-targeted therapies, and continued mechanistic studies with standardized production will be key to advancing MSC-EVs into clinical practice.

## 1. Introduction

Periodontal disease represents a major global public health concern and is recognized as one of the most prevalent chronic inflammatory diseases worldwide [1]. Epidemiological evidence indicates that periodontal diseases affect a substantial proportion of the global population. According to the World Health Organization (WHO) Global Oral Health Status Report (2022), severe periodontitis affects approximately one billion individuals worldwide, underscoring its significant contribution to the global burden of oral diseases [2]. The prevalence of periodontal disease varies considerably across populations, generally ranging from 20% to 50% among adults. These variations are strongly influenced by socioeconomic status, environmental conditions, behavioral risk factors, and access to oral healthcare services [3]. Collectively, these data highlight the widespread and uneven distribution of periodontal disease, reinforcing its status as a persistent global health challenge.

Conventional periodontal therapies, including mechanical debridement and adjunctive antimicrobial treatments, are effective in controlling infection and inflammation; however, their ability to achieve complete regeneration of destroyed periodontal tissues remains limited [4]. The multifactorial and inflammatory nature of periodontal disease, combined with its systemic interactions, underscores the need for innovative regenerative approaches capable of restoring periodontal structures while modulating dysregulated immune responses [5].

Recently, mesenchymal stem cells (MSCs) have emerged as promising therapeutic candidates for periodontal regeneration due to their multilineage differentiation potential, immunomodulatory properties, and ability to promote tissue repair [6]. Although early regenerative strategies focused on the direct differentiation of MSCs into periodontal tissue-forming cells, accumulating evidence suggests that the therapeutic benefits of MSCs are largely mediated through paracrine signaling mechanisms [7,8]. Among these, extracellular vesicles, particularly MSC-derived exosomes, have gained considerable attention as key mediators of MSC-based regenerative effects [9].

In this review, therapeutic potential of MSC-derived exosomes which can regulate inflammatory responses, enhance angiogenesis, and stimulate endogenous tissue regeneration [10] are discussed. The references were identified primarily through PubMed (pubmed.ncbi.nlm.nih.gov), with searches restricted to Title, Abstract, and Keywords. To ensure relevance, a time filter of the last 10 years was applied in most cases, while canonical papers and foundational data were included regardless of publication date. The keywords used to search articles were periodontal disease; mesenchymal stem cell (MSC); paracrine factor; extracellular vesicles (EVs), immunomodulatory. Exosome-based therapies can offer several advantages over cell-based approaches, including improved safety profiles, reduced immunogenicity, and enhanced feasibility for storage and standardization [11]. These characteristics position MSC-derived exosomes as a promising next-generation therapeutic strategy for periodontitis, particularly in patients with systemic comorbidities [12].

## 2. Periodontal Disease as an Inflammatory Burden

The epidemiological patterns of periodontal disease underscore the chronic nature of the disease as well as the inequities embedded in oral health outcomes worldwide [2,13,14]. Beyond its local destructive effects on periodontal tissues, periodontal disease has increasingly been recognized as a condition with systemic implications. Accumulating evidence demonstrates that periodontal disease is strongly associated with a wide range of systemic disorders, including cardiovascular disease, type 2 diabetes mellitus, adverse pregnancy outcomes, and neurodegenerative diseases such as Alzheimer’s disease [15]. The pathophysiological links between periodontal and systemic diseases are primarily mediated through chronic inflammation, microbial dysbiosis, and immune dysregulation, which collectively contribute to systemic inflammatory burden [16,17].

**Figure 1:**
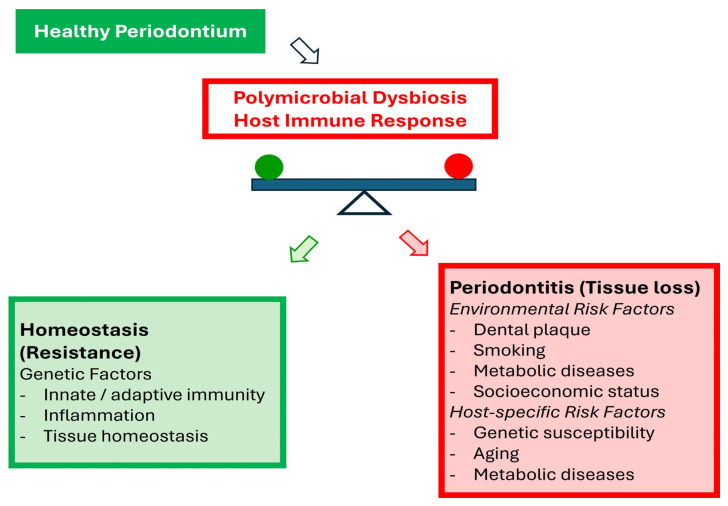
**Conceptual framework of periodontal homeostasis and dysbiosis-driven inflammatory imbalance.** This schematic illustrates the dynamic equilibrium between polymicrobial biofilms and host immune responses in periodontal tissues. In a healthy periodontium, microbial communities coexist with the host in a state of balanced immune surveillance, maintaining tissue homeostasis through coordinated innate and adaptive immune regulation. Genetic background, inflammatory responsiveness, and intrinsic tissue repair capacity contribute to this resistant phenotype. According to the glycation end product (AGE) model, disruption of microbial homeostasis leads to a dysbiotic biofilm that triggers exaggerated host immune activation. Rather than direct bacterial cytotoxicity alone, periodontal tissue destruction is primarily driven by a dysregulated host inflammatory response characterized by sustained NF-κB signaling, excessive cytokine production, and osteoclast activation [20].

Notably, the relationship between periodontal disease and certain systemic conditions, particularly diabetes mellitus, is bidirectional. Diabetes increases susceptibility to periodontal inflammation and accelerates periodontal tissue destruction, while severe periodontitis negatively affects glycemic control and increases the risk of diabetes-related complications and mortality [18]. This complex interplay highlights the importance of integrated therapeutic strategies that address both oral and systemic health [19]. This conceptual framework aligns with the polymicrobial synergy and dysbiosis model and the paradigm of host-mediated inflammatory imbalance in periodontitis (Figure 1), incorporating established environmental and systemic risk modifiers [20]. Collectively, these findings position periodontitis as a prototypical host-mediated inflammatory disorder with systemic consequences, emphasizing that periodontal health is inseparable from overall systemic well-being [17]. At the therapeutic frontier, recognition of periodontitis as an inflammatory burden has spurred interest in host-modulation approaches, including extracellular vesicle-based interventions derived from mesenchymal stem cells (MSCs).

## 3. MSC-EVs as a Novel Regenerative Therapeutic Strategy

The complex bidirectional interactions between periodontal disease and systemic conditions, together with the limited regenerative capacity of current therapeutic approaches, underscore the need for strategies capable of restoring periodontal tissues while concurrently modulating systemic inflammation [21]. Given the chronic inflammatory nature of periodontitis and its reciprocal relationship with systemic disorders, increasing attention has been directed toward regenerative therapies that promote tissue repair while correcting dysregulated immune responses [22].

### 3.1. MSC Therapy for Periodontitis

Mesenchymal stem cells (MSCs) have emerged as promising therapeutic candidates for periodontal regeneration owing to their multilineage differentiation potential, immunomodulatory properties, and paracrine activity [23]. As multipotent progenitor cells, MSCs can differentiate into osteoblasts, fibroblasts, and cementoblast-like cells, thereby contributing to periodontal tissue reconstruction. In parallel, their immunomodulatory capacity—mediated through cytokine secretion, suppression of T-cell proliferation, and regulation of macrophage polarization—enhances their therapeutic efficacy within the inflammatory microenvironment characteristic of periodontitis [24].

Among MSC populations, dental-derived MSCs, including periodontal ligament stem cells (PDLSCs), dental pulp stem cells (DPSCs), and dental follicle stem cells (DFSCs), are particularly relevant due to their origin from cranial neural crest-derived mesenchyme, which naturally contributes to periodontal tissue development (Table 1).

These cells exhibit robust proliferative and differentiation capacities and retain site-specific lineage tendencies, making them highly suitable for translational applications in periodontal defect repair. Notably, DFSCs derived from ectomesenchymal tissues demonstrate strong osteogenic and cementogenic potential and contribute to periodontal ligament fiber formation, differentiating into cementoblasts, fibroblasts, and osteoblasts [30]. In addition, the adult periodontal ligament itself is recognized as a niche harboring neural crest–derived stem cells with intrinsic regenerative potential [25]. Collectively, these findings support a paradigm shift from conventional mechanical therapies toward biologically driven periodontal regeneration [4].

### 3.2. Advantages of EV Derived from MSC

Although early applications of MSCs in regenerative medicine emphasized their direct differentiation into periodontal cell types, accumulating evidence now indicates that their therapeutic effects are mediated predominantly through paracrine mechanisms rather than long-term engraftment or direct cell replacement [31]. In this context, extracellular vesicles (EVs) have gained increasing recognition as key effectors of MSC-based therapies (Table 2).

Extracellular vesicle (EV)–based therapies provide several translational advantages over cell-based interventions. EVs are inherently acellular and non-replicative, avoiding risks associated with living cell transplantation such as uncontrolled proliferation, ectopic tissue formation, or microvascular occlusion [35]. However, the absence of replicative capacity reduces—rather than eliminates—biosafety concerns, as EV cargo composition and potential off-target bioactivity remain incompletely characterized [36].

EVs also offer practical advantages in manufacturing and quality control: they can be produced in scalable culture systems, sterilized by filtration, stored with preserved bioactivity, and standardized using physicochemical and functional release criteria [37]. These attributes support improved batch-to-batch consistency compared with heterogeneous cell populations, although current isolation and characterization platforms do not fully resolve vesicle heterogeneity, and inter-study variability persists [38].

From an immunological standpoint, EVs generally exhibit low immunogenicity and may enable allogeneic, off-the-shelf deployment without donor–recipient matching [39]. Pharmacologically, EVs act as biologically derived nanocarriers capable of transferring proteins, lipids, and nucleic acids across biological barriers. Their small size facilitates tissue penetration and reduces microvascular entrapment relative to intravenously infused cells; nevertheless, in vivo biodistribution and delivery efficiency may be influenced by administration route and microenvironmental context [40]. Systematic long-term immunological evaluations must be done on animals and humans.

Because many cell therapies act predominantly through paracrine signaling, EVs may capture key therapeutic mechanisms while circumventing uncertainties related to cell survival and differentiation fate. In MSC-derived EVs, they carry a diverse repertoire of bioactive cargo, including microRNAs, proteins, and lipids, that modulate inflammatory signaling and enhance regenerative processes [41]. On the other hand, MSC-conditioned medium contains a rich repertoire of soluble factors—antimicrobial peptides, cytokines, and enzymes—that exert bactericidal effects both directly and indirectly (Table 3). These properties also help cell-free therapies in infection control, wound healing, and inflammatory diseases [42,43,44,45,46,47,48,49,50].

Collectively, as cell-free biologics, EV-based therapies offer advantages over cell-based approaches, including improved safety profiles, reduced immunogenicity, greater scalability, and enhanced feasibility for storage, handling, and regulatory standardization [49]. The potential of EV-based therapeutics can be harnessed to bridge fundamental biological insights with clinically relevant applications, providing a platform that combines scalability, safety, and functional versatility. Nevertheless, their successful integration into mainstream medicine will require the establishment of standardized isolation protocols, validated potency assays, and rigorous long-term safety evaluations across diverse patient populations.

Taken together, these attributes position MSC-derived EVs as a rational and feasible therapeutic strategy for periodontal regeneration, particularly in patients with systemically compromised conditions characterized by persistent inflammatory burden [50].

## 4. Mechanisms of MSC-Derived EVs in Periodontal Regeneration

MSC-derived EVs exert their regenerative effects through a combination of immunomodulatory, anti-inflammatory, angiogenic, and pro-regenerative mechanisms that collectively create a favorable microenvironment for periodontal tissue repair [51]. As nano-sized extracellular vesicles (Table 2), exosomes function as key mediators of intercellular communication by transferring bioactive cargos to recipient cells within the periodontal niche [52].

From a mechanistic perspective, the dominant theme centers on osteoimmunomodulation. MSC-EVs promote macrophage polarization toward a pro-resolving phenotype, suppress NF-κB-associated inflammatory signaling, enhance osteogenic programs (e.g., RUNX2/ALP), stimulate angiogenesis, and support matrix remodeling [53]. In metabolically compromised environments, additional benefits include mitigation of oxidative stress and improved reparative signaling.

One of the primary mechanisms by which MSC-derived exosomes promote periodontal regeneration is the modulation of inflammatory and immune responses. Exosomes have been shown to suppress excessive inflammatory signaling by downregulating pro-inflammatory cytokines such as tumor necrosis factor-α (TNF-α), interleukin-1β (IL-1β), and interleukin-6 (IL-6), while enhancing the expression of anti-inflammatory mediators [54]. In parallel, MSC-derived exosomes can regulate macrophage polarization, promoting a shift from the pro-inflammatory M1 phenotype toward the pro-regenerative M2 phenotype, thereby facilitating resolution of inflammation and tissue healing [55]. This involves attenuation of NF-κB-dependent pathways that leads to stimulation of angiogenesis and enhanced osteogenic differentiation as well as macrophage polarization toward an anti-inflammatory M2 phenotype.

MSC-derived exosomes play a critical role in angiogenesis, which is essential for periodontal regeneration. Exosomal cargos, including angiogenic growth factors and regulatory microRNAs, stimulate endothelial cell proliferation, migration, and tube formation, leading to enhanced neovascularization within periodontal defects [56]. Improved vascularization supports nutrient delivery, waste removal, and recruitment of endogenous progenitor cells, all of which are necessary for effective tissue regeneration.

MSC-derived EVs also directly influence the behavior of periodontal resident cells, including periodontal ligament cells, osteoblasts, and cementoblasts [57]. By activating key signaling pathways involved in cell proliferation, migration, and differentiation, EVs promote osteogenesis, cementogenesis, and regeneration of periodontal ligament structures. Moreover, EVs have been shown to enhance extracellular matrix synthesis and inhibit apoptosis, further supporting structural and functional restoration of periodontal tissues [58].

Importantly, MSC-derived EVs can modulate the local microenvironment under pathological conditions, such as hyperglycemia and oxidative stress, which are commonly observed in systemically compromised patients [59]. By attenuating oxidative stress and restoring immune balance, EVs may overcome the impaired healing capacity associated with systemic diseases like diabetes mellitus. Collectively, these multifaceted mechanisms underscore the therapeutic potential of MSC-derived EVs as a cell-free regenerative strategy for periodontal tissue regeneration [60,61,62,63].

## 5. Preclinical Evidence of MSC-Derived EVs

MSC- EVs show potential for clinical translation, demonstrated across multiple animal models [64,65,66,67]. Table 3 summarizes preclinical evidence of MSC-EVs in periodontitis models by aligning model type, functional outcomes, mechanisms, delivery strategies, and sources of variability. Across ligature-induced disease, LPS-driven inflammation, critical-size periodontal defects, and diabetic periodontitis, MSC-EVs consistently demonstrate reduced alveolar bone loss, attenuation of local inflammation, and enhanced periodontal regeneration. In defect models, this includes increased formation of new bone, cementum, and periodontal ligament-like tissue, while in diabetic settings MSC-EVs partially restore impaired healing (Table 4).

Most studies employ local delivery (gingival/periodontal injection or EV-loaded biomaterial scaffolds), reflecting the need to maximize site-specific bioavailability and minimize systemic variability [68]. However, effect size and durability are strongly influenced by EV source (bone marrow, adipose, umbilical cord), isolation and characterization methods that shape cargo composition, dosing schedule, disease stage at treatment, and scaffold–EV interactions. Follow-up durations are often short, and long-term biodistribution, persistence, and safety remain insufficiently characterized.

At present, clinical trials directly evaluating MSC-EVs in human periodontal diseases remain scarce compared with the extensive body of preclinical research, though a few early-stage human studies have begun to emerge. To date, MSC-EV therapeutics have primarily advanced into early clinical testing in non-oral indications (Table 5). Early-phase clinical translation of MSC-EV therapeutics spans neurological, respiratory, gastrointestinal, hepatic, and periodontal indications, predominantly evaluating safety, tolerability, and feasibility across intranasal, inhaled, intravenous, and local delivery routes [64,65,66,67,68]. Moving forward, larger controlled trials and broader applications, including periodontal disease, will be essential to establish optimal dosing strategies, delivery methods, and definitive clinical benefits.

Taken together, MSC-EVs reproducibly couple inflammation resolution with bone and periodontal tissue regeneration in preclinical periodontitis, but translatability is contingent on many factors, including EV source, manufacturing parameters, local delivery design, and well-designed human trials [69]. These considerations highlight both the promise and the challenges of MSC-EVs in periodontal disease, emphasizing that rigorous methodological harmonization, long-term follow-up, and consensus regulatory standards will be essential for their successful clinical translation.

## 6. Limitations of MSC-EVs

Despite their promising regenerative and immunomodulatory potential, mesenchymal stem cell-derived extracellular vesicles (MSC-EVs) face several significant challenges that currently restrict clinical translation. A primary limitation lies in heterogeneity and variability: EV composition is highly dependent on the MSC source (e.g., bone marrow, adipose tissue, dental pulp, umbilical cord), donor characteristics, culture conditions, and passage number [70]. This variability leads to inconsistencies in cargo content—including miRNAs, proteins, and lipids—and contributes to unpredictable therapeutic potency. Batch-to-batch variability remains a critical obstacle for reproducibility and regulatory approval [71].

A second major challenge concerns standardization and characterization. There is no universally accepted protocol for EV isolation, purification, and quantification. Differences in ultracentrifugation, size-exclusion chromatography, precipitation methods, and commercial kits significantly affect yield, purity, and biological activity. Moreover, standardized potency assays that reliably correlate in vitro activity with in vivo efficacy are still lacking, complicating efforts to establish consensus product specifications [72].

Scalability and manufacturing constraints further limit clinical translation. Producing clinically relevant quantities of high-purity EVs under Good Manufacturing Practice (GMP) conditions is technically demanding and costly [73]. Large-scale expansion of MSCs may alter cellular phenotype and EV cargo, diminishing therapeutic consistency. Additionally, storage stability, shelf-life, and preservation methods (e.g., cryopreservation versus lyophilization) require further optimization to ensure product reliability.

Another important limitation involves biodistribution and targeting efficiency. Systemically administered EVs are rapidly cleared by the mononuclear phagocyte system and tend to accumulate in the liver, spleen, and lungs, which may limit effective delivery to target tissues such as the periodontium [40]. Local delivery strategies—such as gingival injections or incorporation into biomaterial scaffolds—can improve site-specific retention but remain challenged by diffusion, degradation, and insufficient tissue penetration. Precise control over tissue-specific targeting remains underdeveloped [68].

Dose optimization and pharmacokinetics are also incompletely defined. The therapeutic window, optimal dosing frequency, and long-term persistence of EVs in tissues remain poorly characterized [69]. Without clear pharmacodynamic profiles, translating preclinical dosing regimens into human trials is difficult. Safety concerns further warrant consideration. Although MSC-derived EVs are generally regarded as less immunogenic than cell-based therapies, their long-term effects remain uncertain. Potential risks include unintended immunomodulation, pro-fibrotic signaling, or tumor-promoting effects in certain microenvironments. In chronic inflammatory settings such as periodontitis, altered host immune responses may unpredictably modify EV behavior [70].

On the other hand, the regenerative effects of MSC-EVs in periodontitis must be compared with current gold-standard therapies, such as guided tissue regeneration (GTR), enamel matrix derivative (EMD) and biomaterials. As robust human trial data on MSC-EVs is lacking and the evidence for their use remains largely preclinical, clinical endpoints such as clinical attachment level (CAL) gain and probing depth (PD) reduction, which are well established for conventional therapies, must be examined in more detail [71,72]. Future clinical trials must therefore establish whether MSC-EVs can achieve reproducible CAL gain and PD reduction, while addressing regulatory and manufacturing challenges.

Finally, evolving regulatory and classification frameworks add complexity. EVs exist at the intersection of biologics, cell-free therapies, and advanced medicinal products, and regulatory requirements differ across jurisdictions [73]. While their acellular nature may facilitate alignment through more defined product composition and reduced biosafety risk, consensus standards for potency assays, product specifications, and scaffold–EV systems are still in development [74]. Biomaterial scaffolds introduce additional layers of complexity, as their physicochemical properties can influence EV release kinetics, stability, and immunological responses [75].

Taken together, these limitations highlight both the promise and the challenges of MSC-derived EV therapy in regenerative medicine. Rigorous methodological harmonization, extended longitudinal studies, and the establishment of standardized potency assays will be essential to ensure safe, reproducible, and effective clinical translation.

## 7. Future Directions

Extracellular vesicles (EVs) are increasingly recognized as central regulators of periodontal pathophysiology, orchestrating intercellular communication within the periodontal microenvironment while extending inflammatory signaling beyond the oral cavity [76]. Both bacterial outer membrane vesicles and host-derived exosomes contribute to immune modulation, tissue destruction, and dissemination of bioactive cargo [77]. By entering circulation and interacting with distant organs, periodontal EVs provide a mechanistic framework linking periodontitis with cardiovascular, metabolic, and other chronic inflammatory diseases [58].

Salivary and plasma EV profiling represents a promising minimally invasive strategy capable of reflecting real-time inflammatory status, stratifying disease activity, and identifying individuals at increased systemic risk [78]. Such liquid biopsy–based approaches may enable earlier intervention, personalized periodontal care, and integrated assessment of systemic inflammatory burden. Therapeutically, pathogenic vesicle signaling may be attenuated, while the regenerative and immunomodulatory potential of MSC-EVs can be harnessed to promote tissue repair [79]. Targeted modulation of EV biogenesis, cargo composition, and tissue-specific delivery may shift the periodontal microenvironment from chronic inflammation toward regenerative resolution, with benefits extending beyond local tissue restoration [80].

For EV-based diagnostics to achieve meaningful clinical translation, rigorous methodological standardization is indispensable. Harmonized isolation and purification protocols, standardized quantification metrics, and reproducibility across laboratories must be established [81]. Large-scale cohort validation studies and prospective longitudinal clinical trials are needed to determine whether salivary or plasma EV signatures can reliably predict disease progression, therapeutic response, or systemic complication risk [82]. At the therapeutic level, the potential of MSC-EVs remains substantial but under-optimized. Enhancing regenerative cargo—particularly osteogenic, angiogenic, and anti-inflammatory miRNAs—represents a critical objective [41]. Surface engineering strategies to achieve periodontal tissue-specific targeting, along with controlled-release delivery systems such as hydrogels, scaffolds, and injectable biomaterials, may improve therapeutic precision and retention [58]. Equally important are dose–response optimization and pharmacokinetic profiling to define therapeutic windows and ensure safety. The integration of bioengineered exosomes with advanced biomaterials may ultimately enable spatially controlled and sustained periodontal regeneration [83].

Future research should adopt a systems medicine framework, integrating oral EV profiling with systemic inflammatory markers, metabolic parameters, and microbiome data to reveal network-level interactions driving chronic inflammatory diseases [84]. High-resolution longitudinal studies combining single-vesicle analysis with multi-omics approaches—including proteomics, miRNA sequencing, lipidomics, and spatial transcriptomics—are required to delineate how EV cargo evolves from gingivitis to advanced periodontitis [85]. Identifying stage-specific EV signatures will enhance mechanistic understanding and enable earlier diagnosis, improved disease stratification, and more accurate monitoring of inflammatory activity.

Further clarification of systemic EV crosstalk is essential. Although periodontal-derived EVs have been implicated in cardiovascular and metabolic disorders, direct causal mechanisms remain incompletely defined. Future studies should track labeled periodontal EVs in vivo to determine biodistribution patterns and organ-specific targeting, while elucidating uptake receptors and endocytic pathways [86].

Successful clinical implementation further depends on robust manufacturing and regulatory frameworks. Scalable GMP-compliant production systems, standardized potency assays, and comprehensive long-term safety evaluations are required [87]. Key considerations include minimizing batch-to-batch variability, ensuring storage stability and shelf-life, assessing immunogenicity within chronic inflammatory environments, and evaluating potential long-term systemic effects. Carefully designed phase I/II clinical trials will be essential to establish safety margins, determine optimal dosing parameters, and meet regulatory standards [88].

Advancing periodontal research toward EV-centered mechanistic and translational paradigms will require interdisciplinary collaboration across dentistry, immunology, bioengineering, and systems biology [16]. Integration of multi-omics data, clinical phenotyping, computational modeling, and longitudinal patient monitoring may facilitate predictive and personalized treatment strategies [89]. Within this system-level framework, EVs function both as mechanistic mediators and actionable biomarkers linking oral and systemic health, ultimately redefining periodontal therapy as a precision-based, systemically informed discipline aimed at improving overall health outcomes [90].

## 8. Conclusions

Extracellular vesicles (EVs) are increasingly recognized as potential mediators in periodontal biology, with growing evidence suggesting that they may function at the intersection of local tissue pathology and systemic inflammatory networks. By orchestrating immune modulation, complement activation, and osteoimmunologic crosstalk, EVs appear to provide a mechanistic link between periodontitis and chronic systemic conditions such as cardiovascular and metabolic disease. Their dual role—as carriers of microbial virulence factors and amplifiers of host immune responses, yet simultaneously as regenerative and immunomodulatory agents—positions EVs as both challenges and opportunities for translational innovation.

From a therapeutic perspective, MSC-EVs hold promise for shifting the periodontal microenvironment from chronic inflammation toward regenerative resolution. Preclinical studies suggest their capacity to restore periodontal tissues, rebalance immune responses, and modulate systemic inflammation [80]. However, significant barriers remain, including heterogeneity in EV composition, lack of methodological standardization, scalability constraints, incomplete pharmacokinetic characterization, and evolving regulatory frameworks [73].

On the diagnostic front, salivary and plasma EV profiling offers a minimally invasive liquid biopsy approach capable of reflecting real-time inflammatory status, stratifying disease activity, and identifying patients at heightened systemic risk [82]. Future progress will depend on rigorous methodological harmonization, longitudinal multi-omics studies, and integrative systems medicine approaches that conceptualize periodontitis not as an isolated oral infection but as a network-driven inflammatory condition interconnected with systemic health [91]. Within this framework, EVs may serve both as actionable biomarkers and therapeutic leverage points, enabling precision-based, systemically informed periodontal care.

Advances in our understanding of periodontal pathogenesis have shifted the therapeutic paradigm from infection control toward modulation of host immunity and promotion of true tissue regeneration. MSC-EVs represent a promising cell-free strategy that integrates immunoregulatory and pro-regenerative functions while overcoming many limitations of direct cell transplantation. However, a substantial translational gap remains despite encouraging preclinical evidence [92]. Current findings are largely derived from in vitro and animal models, and the absence of robust human data limits definitive conclusions regarding efficacy, safety, and long-term outcomes. This underscores the need for carefully designed clinical trials that establish biological plausibility while addressing regulatory considerations, manufacturing challenges (such as GMP compliance for dental EVs), and standardized outcome measures.

In conclusion, continued mechanistic clarification, harmonization of production protocols, and rigorous clinical validation will ultimately determine whether MSC-EVs can transition from experimental innovation to routine clinical application in periodontology.

## Figures and Tables

**Table 1:** Dental-Derived MSCs in Periodontal Regeneration.

Stem Cell Type	Source	Differentiation	Applications	Unique Feature	Ref.
Periodontal Ligament Stem Cells (PDLSCs)	Periodontal ligament tissue	Cementoblasts, fibroblasts, osteoblasts	Regeneration of periodontal ligament fibers, cementum, and alveolar bone	Maintain site-specific lineage commitment; reside in adult PDL niche	[25]
Dental Pulp Stem Cells (DPSCs)	Dental pulp (from permanent or deciduous teeth)	Odontoblasts, osteoblasts, chondrocytes, adipocytes	Dentin-pulp complex regeneration, potential for neurovascular repair	High proliferative capacity; neurotrophic and angiogenic properties	[26]
Dental Follicle Stem Cells (DFSCs)	Dental follicle (ectodermal mesenchymal origin)	Cementoblasts, fibroblasts, osteoblasts	Periodontal tissue engineering, root development support	Derived from cranial neural crest; precursor to PDL fibers	[27]
Stem Cells from Apical Papilia (SCAPs)	Apical papilla of developing teeth	Odontoblasts, osteoblasts	Root dentin and pulp regeneration	High proliferative and migratory capacity; contribute to root formation	[28]
Gingival Mesenchymal Stem Cells (GMSCs)	Gingival connective tissue	Osteoblasts, chondrocytes, adipocytes	Soft tissue regeneration, immunomodulation	Easily accessible; strong immunosuppressive properties	[29]

PDLSCs and DFSCs are most directly relevant for periodontal ligament and alveolar bone regeneration. DPSCs and SCAPs contribute to dentin-pulp complex repair but also show potential in periodontal applications. GMSCs are particularly valuable for their accessibility and immunomodulatory effects, complementing hard tissue regeneration strategies.

**Table 2:** Comparison of different types of extracellular vesicles (EVs).

EV Type	Diameter	Origin	Cargo	Functional Roles	Ref.
Exosomes	30–150 nm	Endosomal origin (multivesicular bodies)	miRNAs, mRNAs, proteins, lipids	Immunomodulation, angiogenesis, osteogenesis, anti-inflammatory signaling	[32]
Microvesicles (MVs)	100–1000 nm	Direct budding from plasma membrane	Cytokines, growth factors, membrane proteins	Cell proliferation, migration, extracellular matrix remodeling	[33]
Apoptotic Bodies	500–2000 nm	Released during programmed cell death	DNA fragments, histones, cellular organelle	Clearance of apoptotic cells, potential immunoregulatory effects	[34]

**Table 3:** Bactericidal Soluble Factors in MSC-Conditioned Medium.

Soluble Factor	Mechanism of Action	Therapeutic Relevance	Ref.
LL-37 (Cathelicidin)	Disrupts bacterial membranes, leading to lysis	Direct antimicrobial activity; useful in infection control and wound healing	[42]
Beta-defensins	Broad-spectrum antimicrobial peptides; permeabilize bacterial cell walls	Enhance innate defense against oral pathogens and systemic infections	[43]
Lipocalin-2	Sequesters bacterial siderophores, limiting iron availability	Restricts bacterial growth; potential in controlling dysbiosis in periodontitis	[44]
Indoleamine 2,3-dioxygenase (IDO)	Depletes tryptophan, impairing microbial metabolism and replication	Immunomodulatory and antimicrobial; balances host defense and inflammation	[45]
Nitric oxide (NO)	Reactive nitrogen species damage bacterial DNA and proteins	Direct bactericidal effect; contributes to MSC-CM antimicrobial potency	[46]
Reactive oxygen species (ROS)	Oxidative stress damages bacterial membranes and intracellular components	Synergistic antimicrobial activity; enhances host immune clearance	[47]
IL-6, IL-8	Recruit and activate neutrophils and macrophages	Indirect bactericidal effect via immune cell activation; strengthens host defense	[48]

MSC-Conditioned Medium provides diversity and rapid action, while MSC-EVs provides precision and stability. Both approaches complement each other in cell-free therapies.

**Table 4:** Representative Preclinical Evidence of MSC-Derived EVs in Periodontitis.

Experimental Model	Principal Functional Outcomes	Dominant Mechanisms Implicated	Route of Administration	Sources of Variability Limitations	Ref.
Ligature-induced periodontitis (rodent, n = 3)	↓ Alveolar bone loss, ↑ bone volume/height, ↓ periodontal inflammation	Osteoimmunomodulation (M1 → M2 macrophage polarization), ↑ osteogenic signaling (e.g., RUNX2/ALP), ↓ pro-inflammatory cytokines (TNF-α, IL-1β)	Local injection (gingival/periodontal), hydrogel-assisted local delivery	Effect size depends on EV source (BM/AD/UC), dose frequency, and disease stage at treatment	[64]
LPS-induced periodontal inflammation (hPDLSCs)	↓ Gingival swelling and inflammatory infiltrate, ↑ tissue repair	NF-κB pathway attenuation, anti-apoptotic signaling, pro-resolving miRNA transfer	Local injection, topical scaffold delivery	Cargo heterogeneity by isolation method; short follow-up windows in many studies	[65]
Periodontal bone defect models (critical-size defects, n = 6)	↑ New bone formation, ↑ cementum/PDL-like tissue regeneration	Pro-angiogenic signaling (VEGF-related), matrix remodeling, recruitment of progenitor cells	EV-loaded biomaterial scaffolds (collagen, hydrogel), local injection	Scaffold–EV interactions not standardized; limited head-to-head comparison vs. MSCs	[66]
Diabetic periodontitis models (n = 3)	Partial rescue of impaired healing, ↓ oxidative stress, ↑ bone regeneration vs. untreated diabetic controls	Redox modulation, enhanced angiogenesis, immunomodulation under hyperglycemia	Local injection, EV–scaffold composites	Metabolic milieu alters EV biodistribution and potency; durability of response unclear	[67]

Periodontitis models consistently show that MSC-EVs couple inflammation resolution with bone/periodontal regeneration via osteoimmunomodulatory mechanisms; however, effect size and durability are strongly conditioned by EV source, manufacturing variables, and local delivery design. Abbreviations: BM, bone marrow-derived; AD, adipose-derived; UC, umbilical cord-derived; PDL, periodontal ligament; hPDLSCs, human periodontal ligament stem cells.

**Table 5:** Current MSC-EV Clinical Trials (https://clinicaltrials.gov/) (Accessed on 14 February 2026).

Trial ID	Condition Indication	Intervention	Delivery Route	Status
NCT04388982	Alzheimer’s disease	Allogeneic adipose MSC-EVs	Intranasal (nasal drip)	Phase 1/2, recruiting
NCT07243561	Autism spectrum disorder	hUC-MSC-EVs	Intranasal spray	Interventional, ongoing
NCT04276987	Severe COVID-19 pneumonia	Adipose MSC-EVs	Inhalation (nebulized)	Phase 1, ongoing
NCT04544215	Drug-resistant pulmonary infection	MSC-EV product	Inhalation (nebulized)	Phase 1/2, ongoing
NCT05127122	ARDS	BM-MSC-EVs (ExoFlo^TM^)	Intravenous infusion	Phase 1/2, ongoing
NCT04657458	COVID-19–associated ARDS	BM-MSC-EVs (ExoFlo^TM^)	Intravenous infusion	Expanded access
NCT05176366	Ulcerative colitis	BM-MSC-EVs (ExoFlo^TM^)	Intravenous infusion	Phase 1, ongoing
**NCT04270006**	**Periodontitis**	**Adipose MSC-EVs**	**Local periodontal delivery**	**Early Phase 1, registered**
NCT05940610	Acute/acute-on-chronic liver failure	MSC-EVs	Intravenous infusion	Phase 1/2, not yet recruiting
NCT06002841	Acute respiratory failure	MSC-EVs vs. placebo	Intravenous infusion	Phase 1/2, pending
NCT05395292	Regenerative/inflammatory indication *	MSC-EVs	Not known (Systemic or local *)	Early-phase, registered *
NCT06825572	Acute / Acute-on-Chronic Liver Failure after Liver Transplantation	MSC-EVs	Intravenous infusion	Phase 1, not yet recruiting
NCT05136885	Regenerative/inflammatory indication *	MSC-EVs	Not known (Systemic or local *)	Early-phase, registered *

* Public indexing for these IDs is limited/variable across registries; fields reflect available registry summaries. MSC-EV therapeutics are transitioning from proof-of-concept to early human trials, with respiratory and hepatic diseases leading the way. Neurological applications are emerging, while periodontal disease remains a promising but as-yet untested frontier in humans. The next few years will likely see Phase II/III expansion and the first dedicated oral/periodontal trials.

## Data Availability

The data presented in this study were derived from the following resources available in the public domain: PubMed (https://pubmed.ncbi.nlm.nih.gov/), ClinicalTrials.gov (https://clinicaltrials.gov/).

## Abbreviations

The following abbreviations are used in this manuscript:

MSC	Mesenchymal stem cell
PD	Periodontal disease
EVs	Extracellular vesicles
